# “If we are waiting for the numbers alone, we will miss the point”: a qualitative study of the perceived rise of food allergy and associated risk factors in the Greater Accra Region, Ghana

**DOI:** 10.1186/s41256-017-0040-0

**Published:** 2017-07-11

**Authors:** George A. Atiim, Susan J. Elliott, Ann E. Clarke

**Affiliations:** 1 Department of Geography and Environmental Management, Faculty of Environment, University of Waterloo, Waterloo, Ontario Canada; 2 Department of Medicine, Cumming School of Medicine, University of Calgary, Calgary, Alberta Canada

**Keywords:** Food allergy, Chronic illness, Epidemiologic transition, Qualitative, Ghana

## Abstract

**Background:**

Globally, food allergy [FA] is considered a growing health epidemic. While much of what is known comes from developed countries, there is growing interest in the epidemiology of FA in developing regions such as sub-Saharan Africa. Indeed, researchers are beginning to document the incidence and prevalence of FA and sensitization. The results outlined in this paper stem from an exploratory qualitative study examining the emergence of the health risk of FA in Ghana, a country undergoing epidemiologic changes.

**Methods:**

Between June and August, 2015, we conducted thirty-seven (37) semi-structured in-depth interviews. This comprised seventeen (17) healthcare workers across 12 public and private hospitals and twenty (20) individuals with FA and families with allergic children. All interviews were recorded and transcribed verbatim. Transcripts were analyzed to develop thematic areas that characterize perceptions and experiences around FA.

**Results:**

Three key broad themes arise from this study. First, FA is an emerging health risk, whose incidence is perceived to be increasing. Second, participants expressed mixed perceptions about the public health burden of FA. Third, participants identified individual and societal factors that may be influencing FA risks and susceptibility.

**Conclusion:**

Our research suggests FA is a growing but unrecognized public health concern. There is the need for health policies and researchers to consider the full extent of ongoing epidemiologic changes for the health of populations in developing regions.

## Background

Allergic diseases including asthma, rhinitis, eczema and food allergy (FA) are a growing global public health challenge [1]. Globally, between 30 to 40% of the world’s population have at least one allergic condition [1, 2] creating serious psychosocial and economic impacts for individuals at risk, their families, and healthcare systems. The interaction between one’s genetics and environmental factors are thought to increase one’s susceptibility and disease occurrence [3]. While the specific pathways remain unclear, researchers suggest a direct relationship between infections and the rise in allergic disease, especially in western societies [4, 5]. For example, a notable review revealed that between 1950 and 2000, as rates of infectious disease declined, the incidence of allergies and autoimmune disease [e.g. multiple sclerosis, type 1 diabetes] increased [5]. Other studies and reviews highlight linkages between exposure to micro organic environments [e.g. living on farms], heightened risk of developing allergies [e.g. asthma, hay fever] and atopic sensitization compared to those living on nonfarm lands or urban places [6–8]. Allergies, therefore appear to follow a gradient along socio-economic status (SES), geographic locality (e.g. rural vs urban) and measures of national incomes. The assumption is that individuals with high SES, an urban lifestyle, and residency in high income countries have increased risk of acquiring allergies and vice versa.

In North America and many parts of Western Europe that have moved through the epidemiologic transition, an *epidemic* of food allergy [FA] has emerged following similar increases in asthma and allergic rhinitis. Studies reveal 7.5% of children and adults in Canada [9], 8% in the United States [10], and 10% in Australia [11] self-report a FA. These rates come with many health and social consequences. For example, FA impose a considerable psychosocial and financial burdens on individuals and their families [12, 13]. Moreover, FA detrimentally shapes one’s quality of life [14] and forces people to constantly negotiate physical safety and social wellbeing [15]. In the most extreme cases, FA can trigger anaphylaxis, a serious life-threatening condition [16, 17]. To date, the only mechanism to manage an anaphylactic allergy is complete avoidance of allergens and use of an epinephrine autoinjector when a severe reaction occurs.

While much of what is known about (food) allergic disease comes from developed countries, there is growing interest in regions such as sub-Saharan Africa (SSA). Especially, given evidence that prevalence of allergic symptoms (e.g. asthma, allergic rhinitis and atopic eczema) have increased in SSA and represent central challenges for child and adolescent health [18], there is growing suspicion that FA will soon be a challenge. Researchers are beginning to ask if countries in Africa will follow the experience of western countries as they complete their health transitions [19–21].

In the absence of population and hospitalization based studies (see 21, 22, 23 for exceptions), a body of small-sample studies are beginning to document the incidence and prevalence of FA and sensitization in SSA. In unselected populations, challenge proven FA was 2.5% in South Africa [22]. In the same context, other studies report high rates of FA – between 18% and 40% [23, 24] and food sensitization - 5% and 66% [24, 25]. In Ghana, self-reported FA and sensitization is estimated at 11% and 5% of schoolchildren respectively [26]. It is important to note that sensitization – *that is a positive response to an immunoglobulin E (IgE) antibody to the offending food* - does not always imply FA. The latter is evaluated partly on the basis of reproducing allergic symptoms [e.g. hives, eczema, shortness of breath, anaphylaxis] upon exposure to an allergen and a confirmation of sensitization to the specific food. In the few recent extant studies and reviews on FA in Africa, the high sensitization rates are increasingly suggestive of clinical symptoms of FA [21]. In addition, they reveal an urban and SES bias in rates of FA and sensitization [27]. Taken together, these studies suggest FA is becoming an important health issue in urban SSA locations. Consequently, there is need for research to understand the changing allergy landscape.

Allergies occur and are experienced differently in various settings. Spatial differences in prevalence is likely mediated by factors such as (but not limited to) variation in access to medical care, culture and language [28]. For example, studies show FA is an unfamiliar health risk among immigrants in Canada [29]. We argue that placing FA within the sociocultural and political environment can provide insights toward understanding the experiences and practices around FA in developing regions. Understanding FA in the context in which they arise will help develop context-dependent responses that address the unique needs of the allergic population.

While available studies that characterize FA incidence and prevalence in the African context use quantitative approaches and markers (e.g. IgE antibodies), they explain very little about the unique ways in which FA is experienced. For example, little is known about local perceptions of FA risk, diagnostic decision-making, management, or the subsequent socioeconomic impact of FA. Qualitative methods are an effective way to describe these health experiences, beliefs, and practices as well as illustrating how processes at multiple levels [30] shape health experiences and outcomes. However, only recently are researchers engaging with qualitative methods to explore FA experiences related to psychosocial responses, management, and coping strategies [31–33]. These studies highlight the importance of understanding how social context affect FA and allergy related behaviours.

In Ghana, the extent of FA is unknown and research to identify and understand the FA front is limited. To focus attention on this health risk, we draw on in-depth interviews with healthcare workers and the allergic population to understand the scope of FA risk in Ghana. Specifically, we ask how do healthcare workers and allergic populations perceive FA and its associated health risks? In so doing, we highlight FA as an emerging public health problem and contribute to the scarce literature on (food) allergic disease in SSA.

## Embodied epidemiology of food allergy

This study employs a lens from ecosocial theory [34], a relational approach to health that characterizes health risk within social and political structures. Ecosocial perspectives to health have been applied in studies that address questions around cholera vulnerabilities [35], race, discrimination, and health disparities [36, 37], maternal health experiences and pregnancy outcomes [38], and the water-health nexus [39].

This paper particularly emphasizes the two core constructs of ecosocial theory, namely *embodiment* and *accountability and agency* in order to understand the health risks of FA. The term embodiment, refers to how people incorporate, biologically, their lived experiences in societal and ecological contexts [34, 39]. Implicit in this construct is how people negotiate, interpret and interact with their environment within particular historical, cultural and political contexts [40]. While embodiment has traditionally focused on the experience of people with disease, we also paid attention to the embodied experience and knowledge of individuals about “normal or abnormal” bodies in order to paint a picture of FA in Ghana.

Accountability and agency focuses on the perceptions and precepts (e.g. definitions, markers, and practices) that shape understandings of FA research and policy. It deliberately draws attention to institutional (e.g. health authorities, media) and individual (allergic persons, physicians) capacity to inform discourses around FA burdens. The debates about the epidemiology of FA – *what is FA? how can it be measured? what treatment strategies are appropriate?* – for example is a testament to the responsibility of everyone (e.g researchers, experts, patients) in exploring our relationship with food, disease, and the creation of FA knowledge [41]. In the context of FA, this construct suggests that while the allergic population (agency) can powerfully affect conditions around health policies, “expert knowledge” (by physicians, allergists) and structural factors (e.g. food and medical-industrial complex; public policy) are more likely to act as either facilitators or barriers [34, 36, 42].

## Research setting and methods

### Study area

**Fig. 1:**
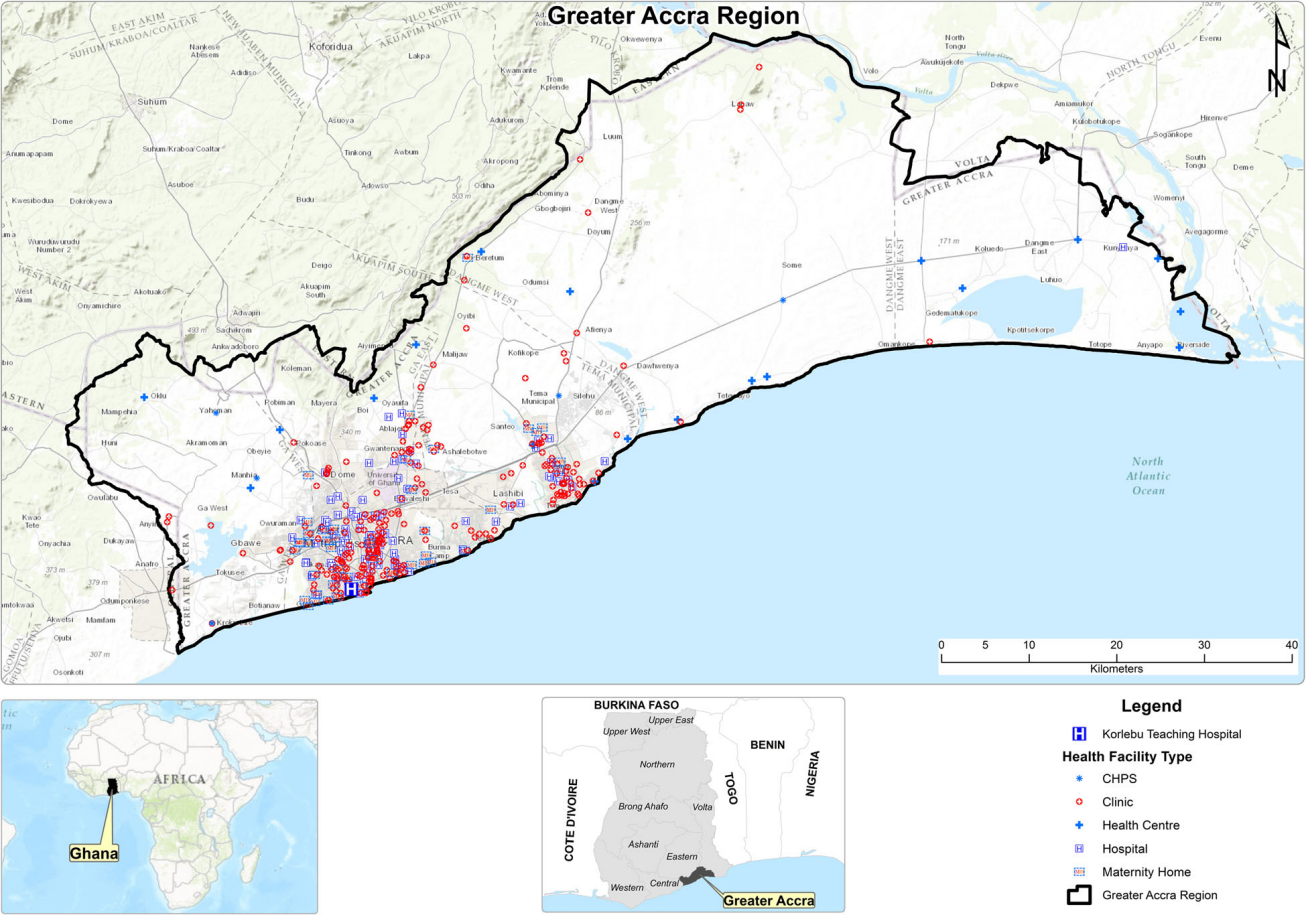
Map of the study area

Ghana is undergoing a rapid epidemiologic transition amidst concerns of growing incidence of noncommunicable diseases (NCDs). For instance, analysis of public and faith-based health facilities data (excluding tertiary hospitals) show an 11-fold increase in outpatient hypertension cases between 1990 and 2010 [43]. Regionally, the majority of reported cases of chronic diseases occur in the Greater Accra Region (GAR) located in south-eastern Ghana [see Fig. 1]. It is one of the most densely populated, and urbanized regions in the country. An estimated 90% of the population reside in urban areas in the GAR [44]. The GAR has one of the highest number of public health infrastructure in the country with 1 regional hospital, 10 district and sub-metropolitan hospitals, 4 polyclinics, 31 health centers and 38 community health and planning service (CHPS) compounds [45]. Consequently, populations in the GAR may have better access, diagnosis and treatment for their health conditions.

Studies show that since the 1950s, chronic diseases have featured as a principal cause of death in the country. For example, cardiovascular diseases (CVDs) rose from being the 17^th^ to 10^th^ leading cause of death by 1966. By 2001, CVDs were considered the leading cause of deaths in the GAR [46, 47]. While the national burden of allergies is unknown, outpatient data suggest a 3-fold increase in asthma cases between 2005 and 2010 [48]. These changing health profiles stem from a combination of factors related to the country’s sustained economic growth, rapid urbanization, increasingly westernized lifestyle (including poor diets) from globalization, and improved healthcare accessibility and utilization.

### Data collection and analysis

Between June and August 2015, we conducted in-depth interviews with seventeen (17) health workers and twenty (20) individuals and families (if child is <18years) with food allergic children in the GAR. For the purposes of this paper, we refer to the latter group collectively as “allergic or affected population” (AP). Interviews provided a means to explore and collect information on a variety of meanings, opinions and experiences with different groups of people [49]. Health workers comprised general physicians (GP), pediatricians (PD), registered dietician nutritionists (RDN), nurses (NR) and traditional health-care practitioners (TP) as well as policy makers (PM). They were recruited in two ways: first, information letters about the study were submitted to heads of health facilities and they informed focal persons at their facility of their interest to participate. Health workers were then contacted by the first author to schedule an interview day and time. Second, we also recruited using snowballing where health workers were encouraged to suggest others who they felt might be interested in this study. Affected persons (AP) were recruited through social media and social networks, flyers (posted at churches, and mosques) and the use of snow ball techniques. If the allergic person was less than 18years old, we interviewed the parent or guardian. To be eligible, participants had to (a) self-reported food allergy or be a parent/guardian of a child with food allergy, or (b) have a physician-diagnosed or suspected food allergy, and (c) must report at least two relevant symptoms, and (d) the symptom(s) must occur within two hours of coming in contact with the allergen. Recruitment continued until no new data emerged (saturation) from the interviews [50]. For health workers and affected persons, saturation was reached on the 14^th^ and 17^th^ participants respectively. We explored participants’ experiences and perceptions related to FA risks, signs, diagnosis, and management.

With respect to health workers, interviews were conducted in an office at the premise of participants’ place of work, at a time of their convenience and lasted between 30 mins and 1 h. For the affected population, most interviews were conducted in their personal residence. All interviews were audio-recorded, collected in the English language and transcribed verbatim for subsequent thematic analysis using QSR International’s NVivo 11. A theme code set was developed both deductively (in line with research objectives and the interview guide) and inductively (themes arising from interview transcripts). Further, inter- and intra-rater reliability for coding [50] was assessed and reached a 90% consensus with a second coder.

Ethical clearance was obtained from University of Waterloo Ethics Review Board (ORE #20670) and the Ghana Health Service Ethics Review Committee (GHS-ERC 02/03/15). In addition, permission was received from administrators of health institutions and our participants.

## Results

Tables 1 and 2 presents a summary of the sociodemographic characteristics of the participants. The major food allergens reported by participants are presented in Table 3 whereas the key clinical symptoms from the perspective of healthcare workers are illustrated in Table 4. In all, three [3] main themes emerged around FA as an emerging health risk, public health significance of FA and FA risk and susceptibility. Results are organized around these themes, and are punctuated by direct quotation from participant interviews.

**Table 1:** Characteristics of healthcare participants [*n* = 17]

Participant characteristics		Number [%]
Sex	Male	6 [35]
Age group	28 – 35	2 [12]
	36 – 43	4 [24]
	44 – 51	3 [17]
	52+	8 [47]
Profession	General Physician [GP]	4 [24]
	Pediatrician [PD]	4 [24]
	Nurse [NR]	4 [24]
	Dietician [RDN]	2 [11]
	Alternative Medicine Practitioners [AMP]	1 [6]
	Public Policy [PPM]	2 [11]
Length of working years	3 – 5	3 [8]
	6 – 10	5 [29]
	11+	9 [53]
Practice setting ^a^	Public hospital services	6 [35]
	Private hospital services	9 [53]
	Hybrid [public & private hospital]	3 [18]
	Public service sector	1 [6]
Districts	Accra Metropolis	5
	Tema Metropolis	5
	Ga West	4
	Ga East	3

^a^ Sum not equal to total number of participants and percentage due to multiple responses

**Table 2:** Characteristics of allergic population [*n* = 22]^a^

ID [PP]	Allergic individual in household				Age of allergic person	Age of diagnosis [DS]	DS	Place of residence	Metropolitan/municipal & district areas
	Child		Self						
	M	F	M	F					
1^x^		X			5	1	PD	La	La municipal
		X			5	1^1/2^			
2				X	25	15		Cantonments	Accra metropolis
3	X				3	1		East Legon	Accra metropolis
4			X		26	18	PD	Tema	Tema metropolis
5	X				12	4		Dodowa	Shai-Osudoku district
6				X	20	11		Adenta	Adentan municipal
7			X		23	17		Weija	Ga South municipal
8				X	25	15	PD	Lashibi	Tema metropolis
9		X			3	1		Dansoman	Accra metropolis
10			X		22	13		Kwabenya	Ga East municipal
11				X	24	10		Sakonono	Tema metropolis
12			X		25	19		Amasaman	Ga West municipal
13^x^	X				12	3	PD	Agbogba	Ga East municipal
		X			8	2			
14			X		25	14		Teshie-Nungua	Ledzokuku-Krowor municipal
15	X				4	1	PD	Osu	Accra metropolis
16			X		23	12		Achimota	Accra metropolis
17	X				5	2		East Legon	Accra metropolis
18	X				4	2		Cantonments	Accra metropolis
19		X			3	1	PD	Ashiaman	Ashiaman municipal
20		X			4	2		Community 2	Tema municipal
Total	12		10						

^a^Participants have more than one child with food allergy

PD – self-report of a physician diagnosis of food allergy

**Table 3:** Most frequently reported or presented food allergens at healthcare institutions

Food allergen	Healthcare workers		Allergic population	
	# of participants [*n* = 17] ^a^	% of participants ^a^	# of participants [*n* = 20]^a^	% of participants^a^
Peanut	10	59	8	36
Fruits (e.g. pineapple)	7	41	9	41
Seafood (e.g. fish, shrimps, prawn)	6	35	6	27
Vegetable (e.g. kontombire)	5	29	5	23
Tubers (e.g. yam, cassava)	4	24	3	14
Sesame	3	18	-	-
Egg	3	18	4	18
Milk	3	18	3	14
Cowpea	2	12	2	9
Soy	-	-	2	9
Corn	2	12	1	3

^a^ Sum is not equal to number of participants and percentage due to multiple responses

**Table 4:** Healthcare workers report of clinical signs and symptoms of food allergies

Description of symptoms	# of mentions [%]	# of respondents mentioning [*n* = 17, %]
Skin/Cutaneous		
Itching	21 [41] ^a^	12 [71] ^b^
Rashes	14 [27]	9 [53]
Hives	9 [18]	7 [41]
Swells [e.g. face, lips, eyes]	7 [14]	5 [29]
Gastrointestinal		
Diarrhea	18 [51]	15 [88]
Vomiting	11 [31]	10 [59]
Abdominal pains	6 [17]	6 [35]
Respiratory		
Breathing difficulties	12 [41]	7 [41]
Persistent cough	8 [28]	4 [24]
Swelling [e.g. tongue, throat]	5 [17]	3 [18]
Running nose	4 [14]	2 [12]

^a^This represents the number of mentions of sign/symptom, and percentage of total mentions; ^b^Number of participants mentioning sign/symptom and percentage of total participants

### An emerging health risk

There was a high level of consensus among most healthcare workers [*n* = 15] and allergic population [*n* = 18] that food allergies are a “new” health phenomenon that is in “its early stages “or “just beginning” or ‘springing up”. Drawing on past experiences, many participants perceived that food allergies were absent in the past but becoming an emerging issue.

I began to deal with these issues only in the last 5 to10 years. I have been working since the 90s and this wasn’t an issue we treated [GP 1, public hospital].

They are certainly very common these days than when we were children. It did not exist those days because none of us had a food allergy [PP 9, mother of allergic child]

On the contrary, some participants felt that food allergy is an old but unrecognized health problem. These participants acknowledged that poor health seeking behaviours may have contributed to under-reporting of FA in the past and consequently the perception that FA is a new phenomenon.

It has always existed. I think the problem is people often don’t report these issues [AMP 1]

Further, most participants, especially healthcare workers [*n* = 13] suggested an observed increase in the incidence of children and adults reporting allergic reactions to food at health facilities.

There is a rise in the frequency of new cases. We used to get one case out of a hundred people referred here. But now we are seeing an increase in the number of people coming. So there is a rise in the frequency of new cases [RDN 1, public hospital]

Many parents are bringing their children. Every now and then, you are treating a case of food allergy. The numbers coming are higher now than before [GP 2, private hospital].

Interestingly, we observed apparent differences between public and private healthcare participants. Though public healthcare workers felt incidence was increasing, they were less likely to report any rise in new cases of FA. On the other hand, their counterparts in private institutions were more frequently reporting a rise in new cases of FA.

However, healthcare workers were unanimous in their perception of FA prevalence. Many acknowledged that the lack of baseline data (i.e. no national prevalence data) and an inadequate disease surveillance system (i.e. existing system does not capture FA cases) were a barrier to gauging the prevalence of FA in the country.

We don’t have national data on it. And that’s the problem. There are no surveys to tell us how many people have a food allergy [GP 2, private clinic].

The current system we have does not record food allergy cases. It is mostly surveilling the regular ones; cholera, malaria, hepatitis, TB and others. Without surveillance data, it will be difficult to tell whether prevalence is increasing or not [PPM 1].

Notwithstanding this, when asked to speculate, the majority of participants [89%] felt that prevalence of FA is lower, less than “one percent” of the population. Other participants compared FA prevalence to the existing burden of infectious disease:

It’s difficult to estimate but my guess is it has not reach the level we are seeing for the infectious ones. I think it is very much lower [GP 4, public sector]

Allergic participants however perceived that the prevalence of food allergies were increasing. Most participants [*n* = 15] articulated that allergies are more common in recent times especially among children and in school-based settings.

I think this is increasing. There are many children in his school who also have food allergies. They are really very common [PP 13, mother of allergic boy]

Many of my friends also say their kids have food allergies. They tell me when I give him this, he throws out or gets hives and things like that. So is like a lot of children these days have it [PP 18, father of allergic girl]

Overall, the examples illustrate FA as an emerging public health concern whose incidence is perceived to be increasing. The characterization of FA as “common” and “new” highlights the need to pay attention to ongoing changes of the health of the population. At the same time, they also suggest the need to unpack factors within the socio-cultural environment that act as barriers to understanding FA prevalence.

### Perception of the public health importance of food allergy

Results indicate that, most healthcare workers [*n* = 14] felt FA was not a major public health problem of great concern. A key criterion for assessing the public health significance of FA was the relative size and impact of FA compared to infectious disease:

FA is not a big public health issue at the moment. If you look at the last 5 years, it has never been a top 10 cause of visits or admissions compared to malaria and the other chronic ones [PD 3, private clinic].

It does not reflect in attendance to clinics. It’s not an issue we should be worried about. Any public health issue must affect up to 5% of the population and I don’t think we are there yet with food allergy [GP 4, public hospital].

On the contrary, others [*n* = 3] also articulated that “the risk” or “potential” negative outcomes should be the consideration for measuring the public health significance of FA. Physiological (e.g. anaphylaxis) and psychosocial impacts (e.g. quality of life) were key concerns of participants:

We know it can cause death and anything that can lead to death is a serious issue. So the numbers are important but if we are waiting for the numbers alone, we will miss the point [NR 4, private clinic]

It already affects people and its changing lives. When you deal with parents whose child struggles with this, they are always frustrated and anxious. They are worried because they fear the unknown [PD 4, private clinic]

Such perspectives were pervasive in discussions of the public health significance of FA with the allergic population. Some participants [*n* = 12] acknowledged that FA was not given the same importance as other health challenges, with many alleging that FA is not often taken seriously [*n* = 9] in the community. In calling for public health focus, an allergic individual intimated:

Something bad should not happen before they start taking this issue seriously. This thing is uncomfortable. You miss school because of that and the dangerous part is you can also die when it gets serious. It’s time they made this a major issue [PP 2, girl allergic to fish and peanut]

For parents of allergic children, the lack of a health system focus on FA was seen as arising from a poor understanding of the impacts of FA on work – related productivity.

People are affected at their job places. So they should look at it from this point. People are missing or leaving work to take kids to clinic or look after them. Is this not affecting productivity? [PP 1, father of allergic girls]

### Factors influencing FA risk and susceptibility

Two main interrelated sub-themes emerged in discussions around FA risk: risk populations (who is susceptible?) and factors or causes of FA (what is driving this?). Most healthcare participants believed infants and children, people from high socioeconomic groups (e.g. “the educated”, “people who have money”) and place of residence (e.g. living in an urban area) were the most vulnerable to developing FA. Indeed, participants frequently characterized clients seeking healthcare as belonging to the “middle” or “high class” or “those with higher education” as illustrated in this example:

They are mostly children. Those from middle class families. I mean with the degrees, the white collar jobs and often with behaviours akin to western societies [NR 2, private clinic]

Both healthcare workers and allergic population also identified factors at the individual [e.g. genetics] and societal level [e.g. globalization of foods, new methods of food production, urbanization] that influenced population vulnerability to FA risk. At the individual scale, most participants believed a genetic predisposition may increase risk of developing FA:

We know it’s more of a genetic issue. It’s more inherent and so if a parent or family member has any allergies, it is very likely one of the children will have one allergy or another [PPM 1]

I was told you don’t get it the way you get like malaria or TB. They say it’s usually a family thing. They said she has it because her dad suffers from asthma [PP 20, mother of allergic girl]

Also, most participants acknowledged that risk and susceptibility increases as societal changes become apparent. For example, a key concern was the globalization of foods. Participants believed the change from local to baby formula foods had compromised the immune system of children and as result “modern day” children are more susceptible to FA:

The foreign baby foods I believe are one of the reasons. No one feeds babies with local foods these days. Is all about “cerelac” and baby formula foods. It is not surprising that; a lot of babies today have bodies (immune system) that are not very strong [NR 1]

We all grew up eating local porridge and local foods as kids but that’s all changed today. We have been quick to switch to many of the foreign food varieties. Maybe that is why these things are coming up lately [PP 11, allergic female]

Others identified behavioural and lifestyle changes as a contributory factor of FA emergence. Participants acknowledged that changes such as reduced frequency of personal cooking [*n* = 9], eating from outside sources [*n* = 6], and storage of food [*n* = 2] over time has increased the risks of having a FA.

I think it’s because we are cooking a lot less these days and doing a lot more eating outside. The food is not fresh. We come into contact with new things that our system does not like [PP 14, allergic female].

We have copied everything, trying to live western life. We have introduced alien foods in our diets. We are refrigerating and storing more food than we did before. The end result is we are also getting their health issues. If you are copying everything, that is what you will get [RND 4]

Overall, these varied accounts highlight participants’ perceived links between broader changes in the social environment and FA risk.

## Discussion

Embodiment and accountability help us to understand the relation between ongoing socio-environmental processes and changing disease profiles, and health outcomes in Ghana [34, 39]. They draw attention to how bodies negotiate and interact to characterize experiences of health. By drawing on the perceptions and experiences of healthcare workers’ and allergic populations, this study highlights three main characteristics of FA health risk while hinting of the need to pay attention to the role of broader contextual factors (e.g. data availability, absence of FA surveillance system) that shape the understandings of FA in a developing country context.

First, we show that in a context where infections and chronic conditions are considered to be “established” health problems in the healthcare system, FA is becoming an important health issue. This is important, especially given the tendency in the theoretical and empirical literature to implicitly assume the rarity of FA in SSA. Participants suggested that FA might be in its “initial phase” in Ghana. This was evident in the frequent use of phrases such as “it’s just beginning”, “now coming up”, or “springing up” to describe FA risk. This result is consistent with recent reviews that suggest FA is an emerging health problem that is increasing in Africa [21] and Asia [51]. FA has largely been linked to the effects of exposure to microbes, increased hygiene, lack of vitamin D, genetics, dietary fat, caesarean section, obesity and affluence [52, 53]. In short, FA results from changes in our environment. The perception that FA is an emerging health risk suggests the environmental triggers of FA may already be underway in countries like Ghana that are experiencing rapid social, health and economic changes.

Tables 3 and 4 show the most common food allergens and symptoms reported. Prior studies show differences in allergens in developing countries. For example, whereas beans, fruits and peanuts predominate in Ghana [26], in South Africa and Colombia, eggs, peanuts, fruits/vegetables, seafood and meat respectively are the most common [24, 54]. In Ghana, national data do not exist, and the identification of peanut, a major trigger in 60–80% of anaphylactic reactions in western societies [16, 17] raise serious public health challenges. Beyond, peanut being a major ingredient in local diets and nutritional value (vitamins & minerals), peanut, tree-nut and seafood allergy tend to persistent, and a recurring issue in adult life [52] with serious socioeconomic, healthcare, and management implications. We also found that local allergens (e.g. tubers, corn) are important sources of allergic reactions, highlighting the need to pay attention to the role of novel foods such as legumes, potato, sesame etc [55, 56]. While reports of symptoms may suggest food allergy, in the absence of testing, uncertainty remains whether these symptoms are immune-mediated especially the possibility that some symptoms (e.g. itching, water eyes, running nose) could also be induced by other irritants (e.g. pollen, dust mites, pets).

This study also reveals differences in the perception of FA incidence between public and private healthcare workers. The latter frequently indicated increases in new cases of FA than the former. Given the availability of universal health coverage regardless of one’s status in Ghana, we think an increase in the use of private healthcare services by the allergic population may explain this difference. Public health services are often perceived to be of low quality with deficiencies in the provision of essential supplies and infrastructure compared to their private healthcare counterparts [57, 58].

Second, we also found mixed perceptions of the public health significance of FA. Most especially health workers perceived that FA does not yet represent a major public health problem. They suggested because FA is not a major cause of hospitalization and its extent is unclear, expectations of a public health response was consequently unrealistic. This raises important questions around the role of lack of data, under-diagnosis and health priority setting in mediating the distribution of FA. For example, how does FA “become” a major public health problem when there are no attempts at counting it? While the assertion that FA is not a regular feature in healthcare settings may reflect lower rates, given that there are no reliable data, it is possible FA may be occurring at the periphery of the formal healthcare system. Indeed, studies of diabetes in Ghana suggest that patients often seek care from traditional health services first before utilizing hospitals [59]. Other factors such as levels of awareness and familiarity, access, availability, and costs associated with health service utilization may also affect documentation of FA incidence [57, 60, 61].

Following this, the study hints of the need to focus on the sociocultural context in understanding perceptions and experiences around FA. In Ghana, the focus of integrated disease surveillance strategies continues to target infectious disease [62]. Even in the context of NCD prevention, FA is still a neglected condition. Participants’ assessment of FA in the context of the current burden from communicable and NCDs for example raise an important question around “who is responsible for the occurrence of embodiment”? [63]. In a context of limited resources, and uncertainty over the magnitude of FA, policy makers face a dilemma over where to place priorities in terms of health investments. However, given the psychosocial burden of FA and the fact that FA is a high risk fatal health problem, we think FA rates do not have to be comparable to those of infectious or other NCDs in order to trigger a public policy response. There is need to find ways of balancing health priorities to protect the needs of the allergic and general population.

Third and lastly, the findings also illustrate participants’ ability to connect FA risk to ongoing socio-environmental changes. In identifying FA production, participants blamed the emergence of FA on a number of factors, including rising incomes, improved education, growing rates of urbanization, increased adoption of westernized lifestyles and genetics. While studies examine knowledge, perceptions and attitudes towards FA, diagnosis, management, and capacity needs of health workers [64, 65], with few exception [66], perceptions around FA risks and etiology remain largely underexplored. In the context of ‘new’ health risks, understandings perceptions around the etiology and drivers of FA is critical to starting conversations around measuring and managing FA.

Addressing data gaps and incorporating FA into disease surveillance is an important first step to understand and obtain a complete picture of FA, its associated risks and underlying causes. Second, it is critical to informing the development of appropriate policies and initiatives to address FA. However, discussions with participants in this research lead us to believe that nobody will count it until it becomes a major public health problem. In short, decision makers are still waiting for the numbers. But as the history and experience of the rise of NCDs in developing countries suggest, we cannot adopt a “wait and see” reactionary approach to rapidly emerging health risks such as FA.

To address data gaps, we have suggested elsewhere for the inclusion of validated FA questions into existing population-based projects (e.g. Demographic and Health Surveys; Multiple Cluster Indicator Surveys) to begin measuring prevalence and to understand its distributions [19]. In addition, health promotion initiatives on the symptoms and signs associated with FA can give much needed visibility to this emerging health risk. The expectation is that such increased awareness in concert with the removal of barriers to health care would lead to more encounters between the healthcare system and allergic individuals.

This study has limitations. The small sample restricts generalization though providing a rich, diverse and nuanced accounts of FA in Ghana. Also, we were unable to confirm from medical records, self-report or self-report of physician diagnosis of FA. In the absence of gold standards of testing FA in the country, we cannot be certain of the allergic status of individuals. As a result, our findings should be interpreted with caution.

## Conclusion

To our knowledge, this is the first study to qualitatively explore health workers’ and allergic population perspectives of FA in Ghana and the broader African context. Only one study [67] has quantitatively explored knowledge and practices of health professionals in healthcare setting in Africa. By illustrating perceptions and experiences, a nuanced understanding of FA in Ghana is provided. While we recognize there are many debates in the literature regarding the epidemiology of FA, it is important to note that current “consensus” about FA have been shaped by the experiences and concerns of researchers, clinicians, the allergic population and advocacy groups [41, 42]. By focusing on health workers and allergic population, this study highlights valuable knowledge that can help shape our understanding of FA in the “developing” world. We believe such context-based information are meaningful to “shed light on the social processes at play in the emergence of new epidemic” [42].

Our study also suggests that health in the so-called “developed” and “developing” region is beginning to share more similarities than differences. As most parts of the developing world experience rising burdens of NCDs, and evidence emerge about common risk factors with allergic disease, FA can no longer be framed as an isolated disease of western societies. Policy makers therefore need to consider the full scope of the implications of the ongoing social, economic and environment changes for the health of populations in developing countries. This study provides a starting point in shining a spotlight on a growing public health problem.

## Abbreviations

CVD: Cardiovascular disease
FA: Food allergy
GAR: Greater Accra Region
NCD: Noncommunicable disease
SSA: Sub-Saharan Africa
